# Prevalence and Risk Factors of Oral Lesions in a Portuguese Subpopulation: A Retrospective Study

**DOI:** 10.3390/jcm14103294

**Published:** 2025-05-09

**Authors:** Carolina Doroteia, Gonçalo Martins Pereira, Luís Proença, José João Mendes, Maria Alzira Cavacas

**Affiliations:** Egas Moniz Center for Interdisciplinary Research (CiiEM), Egas Moniz School of Health & Science, 2829-511 Caparica, Almada, Portugal; gpereira@egasmoniz.edu.pt (G.M.P.); lproenca@egasmoniz.edu.pt (L.P.); jmendes@egasmoniz.edu.pt (J.J.M.); mariaalziracavacas6@gmail.com (M.A.C.)

**Keywords:** oral–systemic disease(s), prevalence, oral cancer, risk factors

## Abstract

**Background/Objectives:** Oral cancer is a significant global public health concern. Understanding the prevalence and associated risk factors of oral lesions is essential for developing effective preventive strategies. This study aimed to assess the prevalence and risk factors of oral lesions submitted for biopsy in a Portuguese subpopulation. A retrospective cross-sectional analysis was conducted using data from patients who sought dental care at the Egas Moniz Dental Clinic (EMDC) in the Lisbon metropolitan area. **Methods:** Data analysis was performed on a sample of 264 patients who attended the EMDC between October 2016 and December 2019 to ascertain the presence of oral lesions, their different types, and their correlation with potential risk factors. The analysis included all patients who underwent biopsy, and their pathology reports were reviewed. **Results:** The prevalence of oral lesions was 10.3%, affecting 58.7% females and 41.3% males, with a mean age of 55 years. The most frequently biopsied site was the buccal mucosa (23.5%). Non-neoplastic lesions accounted for 75.0% of cases, while mesenchymal lesions were the most common neoplastic category (58.5%). The most prevalent diagnosis was fibroepithelial hyperplasia (36.7%). A statistically significant association was observed between neoplastic lesions and statin use, as well as between both neoplastic and non-neoplastic lesions and the use of antidiabetic medications. **Conclusions:** Oral lesions are prevalent, with non-neoplastic lesions being the most frequent. Understanding their nature, prevalence, and associated risk factors is crucial for early and accurate diagnosis, aiding in disease prevention and management.

## 1. Introduction

The oral cavity comprises various tissue types with different embryological origins and physiological functions, which are closely interrelated. Consequently, the oral mucosa is susceptible to developing a range of reactive, inflammatory, infectious, immune-mediated, and neoplastic conditions [1].

Oral cancer is a significant global public health concern, ranking among the ten most common cancers worldwide [2]. In 2022, the World Health Organization (WHO) estimated more than 389,000 new cases and 188,000 deaths from oral cancer [3]. In Portugal, 1293 new cases and 428 deaths were reported. The WHO predicts a 14.4% increase in new cases of oral cancer in Europe and a 7.6% increase in Portugal by the year 2040 [4].

In addition to malignant neoplasms, the oral cavity is also affected by a wide range of benign and potentially malignant lesions, which are often underrepresented in the literature. Benign oral lesions account for a substantial portion of mucosal pathologies and may cause pain, discomfort, or functional limitations, interfering with patients’ quality of life—particularly in relation to mastication, speech, and swallowing [5]. Despite their clinical relevance, epidemiological studies focusing on benign oral lesions remain limited, especially when compared to the wealth of data on dental caries and periodontal disease [6]. Furthermore, most available studies analyze lesion distribution based on specific age groups, etiologies, or risk factors, with only a few addressing these pathologies in broader, unsegmented populations [7].

Oral potentially malignant disorders are defined as a group of mucosal conditions with an increased risk of malignant transformation. These include leukoplakia, erythroplakia, erythroleukoplakia, oral lichen planus, oral submucous fibrosis, and oral epithelial dysplasia, each with distinct clinical and histopathological features and etiologies [5]. The early identification and appropriate management of these disorders are essential for preventing progression to oral squamous cell carcinoma [8]. A recent systematic review estimated the global prevalence of oral potentially malignant disorders at 4.47% (95% CI: 2.43–7.08), reinforcing the importance of these conditions within oral health research and public health policy [9].

On the other hand, oral cancer is associated with various risk factors identified by the WHO, including tobacco and alcohol use, poor nutrition, and specific viral infections such as human papillomavirus (HPV) [10]. The 17 Sustainable Development Goals (SDGs) were established in the United Nations’ Agenda 2030 action plan to promote global peace, prosperity, and well-being. Specifically, SDG 3 aims to ensure healthy lives and well-being for all. Indicator 3.4 sets the goal of reducing premature mortality from non-communicable diseases by one-third by 2030 through prevention, treatment, and mental health promotion [11]. Although oral health is not explicitly mentioned in SDG 3, research indicates a strong link between oral and overall health, as both share common risk factors [12]. Implementing oral health care and promotion strategies could contribute to overall health improvements, reinforcing oral health as a key component in achieving SDG 3 [13].

To the best of our knowledge, this is the first study estimating the prevalence of oral lesions and their associated risk factors in a population sample from the Lisbon Metropolitan Region. This study aims to assess the prevalence of oral lesions and their association with sociodemographic and behavioral risk factors in an adult Portuguese subpopulation from the country’s most populous suburban area. The findings address the existing data gap for this condition in the region and contribute to the development of effective management and prevention strategies.

## 2. Materials and Methods

### 2.1. Study Design and Eligibility Criteria

This retrospective cross-sectional study analyzed patients who attended the Egas Moniz Dental Clinic (EMDC) between October 2016 and December 2019. The EMDC, located in the municipality of Almada in the Setúbal Peninsula (a NUTS III sub-region within the NUTS II Lisbon region), provides dental health services to the public.

NUTS refers to the Nomenclature of Territorial Units for Statistics, a system used by Eurostat to classify regions within the EU. In the 2024 revision, Portugal is organized into three levels: NUTS I (macro-regions), NUTS II (nine regions, including Península de Setúbal as a newly independent region), and NUTS III (26 sub-regions). Península de Setúbal, where the municipality of Almada is located, is now classified as both a NUTS II and NUTS III region.

During the initial appointment, patients underwent dental triage, which included completing a validated health and lifestyle questionnaire, as well as oral examinations and radiographic assessments to determine their treatment needs. Patients with suspected oral lesions were referred for an oral medicine consultation, during which they were evaluated and, in certain cases, underwent a biopsy. Clinical examinations were performed by experienced dentists, ensuring a high standard of clinical judgement. Histopathological diagnoses were always made by the same pathologist, with all slides subsequently reviewed by a second pathologist to ensure diagnostic accuracy and consistency.

To be included in the study, patients had to be capable of understanding and signing the informed consent form and possess an anatomopathological report of the lesion. This study was approved by the Egas Moniz Ethics Committee and adhered to the Strengthening the Reporting of Observational Studies in Epidemiology (STROBE) guidelines.

### 2.2. Data Collection and Variables

Prior to the clinical examination and radiographic assessment, all patients completed a general health, oral health, and lifestyle questionnaire, which included information on age, gender, employment status, smoking habits, drug use, alcohol consumption, systemic diseases and medications, use of oral prostheses, previous oncological history, and oral hygiene habits. Patients suspected of having an oral lesion were referred for an oral medicine consultation, where a biopsy was performed, and an anatomopathological report was generated.

Out of 13,281 individuals who attended their first consultation at the university dental clinic during the study period, 1361 were referred to the Department of Oral Medicine, and 336 underwent a biopsy during their consultation, making them eligible for this study. Of these, 72 participants were excluded due to incomplete questionnaires, resulting in a final sample size of 264 individuals, corresponding to 264 biopsies.

Age was categorized into three groups: 20–44 years, 45–64 years, and 65 years or older. Employment status was classified as employed, unemployed, student, or retired. Smoking habits were defined as non-smoker or smoker, with smokers further classified as light (<10 cigarettes/day), moderate (10–20 cigarettes/day), or heavy (>20 cigarettes/day). Oral hygiene habits were assessed based on toothbrushing frequency and categorized as 2–3 times daily, once daily, or 2–6 times per week. All systemic diseases and medications reported by the participants were recorded and included in the study. All medications reported by participants were categorized using the Anatomical Therapeutic Chemical (ATC) classification system at levels 2 and 3 [14]. This classification enabled a standardized and unbiased grouping of drugs into pharmacological classes, facilitating a more structured analysis of potential associations between medication use and oral lesions. Diagnoses were categorized into three groups for analysis: non-diagnostic, non-neoplastic lesions, and neoplastic lesions. Neoplastic lesions were further classified into three major subcategories: epithelial, mesenchymal, and hematologic. Lesions were classified according to the latest classification of the International Agency for Research on Cancer [15], and their topography was based on the International Statistical Classification of Diseases and Related Health Problems (ICD-10) [16].

### 2.3. Data Analysis

Data analysis was performed using IBM SPSS Statistics v. 29.0 (IBM Corp., Armonk, NY, USA). Categorical variables were expressed as frequencies and percentages (%). Possible associations between oral lesion groups and different variables were evaluated using the chi-square test and Fisher’s exact test. The level of statistical significance was set at 5%.

## 3. Results

The sociodemographic, behavioral, biometric, health and oral hygiene data of the sample studied are detailed in Table 1. The prevalence of oral lesions in this subpopulation was 10.3%. Of the 264 patients included, 57.7% were female and 42.3% were male. Age ranged from 20 to 89 years, with oral lesions being more prevalent in individuals aged 45–64 years (38.3%). Regarding employment status, 44.3% of the subjects were employed and 34.8% were retired. Non-smokers comprised 73.5% of the sample, and, among active smokers, light smokers comprised the majority (45.7%). Most subjects in the sample (65.2%) had systemic diseases such as hypertension (34.5%) and diabetes (11.7%).

The most common oral site affected was the cheek mucosa (*n* = 62), followed by the gingiva (*n* = 59), lip (*n* = 34), tongue (*n* = 27), palate (*n* = 23), maxilla (*n* = 22), mandible (*n* = 18), oral vestibule (*n* = 6), floor of the mouth (*n* = 5), retromolar area (*n* = 4), and oral commissure (*n* = 4).

In our study, 59.8% of the subjects were regular medication users. Of these, 33.7% reported using antihypertensives (C09), 15.2% used statins (C10AA), 12.1% used antidepressants (N06A), 10.6% used antidiabetics (A10), 2.7% used non-steroidal anti-inflammatory medications (M01A), and 2.7% used analgesics (N02).

**Figure 1:**
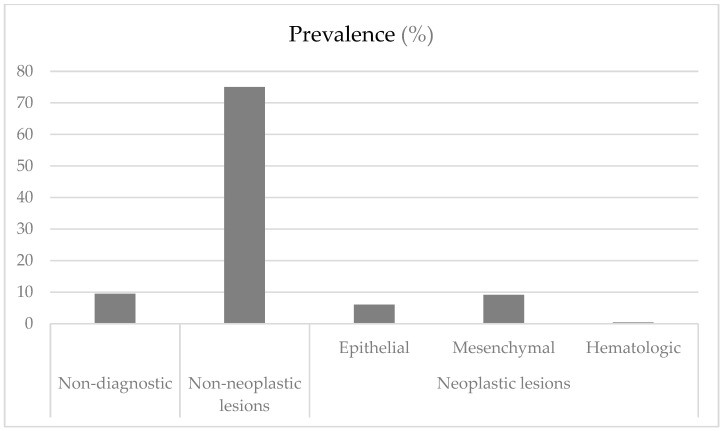
Prevalence of oral lesions presented as groups.

Figure 1 shows the division of the diagnosis into three groups: non-diagnostic, non-neoplastic lesions, and neoplastic lesions. Of the 264 lesions analyzed, 198 (75.0%) were non-neoplastic, 41 (15.5%) were neoplastic, and 25 (9.5%) had no definite diagnosis. Among the neoplastic diagnoses, mesenchymal lesions were the most common (9.1%).

### 3.1. Non-Neoplastic Lesions

The most common lesion was fibroepithelial hyperplasia (n = 97), followed by inflammatory odontogenic cyst (n = 19). Table 2 shows that these lesions were more frequently observed in females and individuals aged 45–64 years (n = 81). As for active smokers, they comprised 26.8% of the population, with the majority being light or moderate smokers (41.5%). Around 63.1% (n = 125) had systemic diseases, and 58.6% (n = 116) were taking medication, with 53.4% of them being polymedicated. Most individuals (66.7%) did not use dental prostheses.

Among the non-neoplastic lesions, histopathological diagnoses suggestive of potentially malignant behavior included hyperkeratosis (n = 13), features compatible with oral lichen planus (n = 13), and epithelial dysplasia (n = 3).

### 3.2. Neoplastic Lesions

Males were slightly more affected (n = 21) compared to females (n = 20). Neoplastic lesions were most frequently observed in individuals aged ≥ 65 years (n = 18). Approximately 24.4% of individuals were active smokers, and, of these, the majority were light smokers (70.0%). Around 70.7% (n = 29) had systemic diseases, and 58.5% (n = 24) were taking medication, with 50.0% of them being polymedicated. Most individuals (65.9%) did not use dental prostheses. Within this group, the most common lesions were squamous papilloma (n = 12) and hemangioma (n = 12).

Regarding malignant neoplasms, two individuals were diagnosed with squamous cell carcinoma, both with a history of diabetes and the use of antidiabetic medications. This lesion was found in both males and females, one on the tongue and the other on the gingiva.

**Figure 2:**
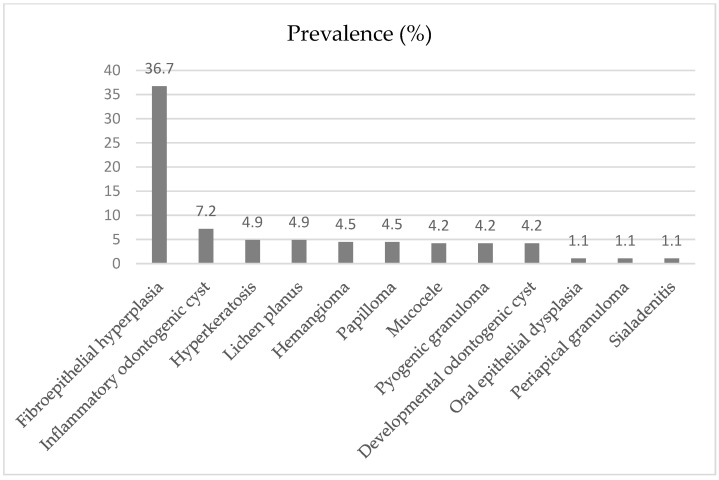
Prevalence of 12 most common oral lesions and conditions.

Figure 2 shows that fibroepithelial hyperplasia (n = 97) was the most common diagnosis, followed by inflammatory odontogenic cyst (n = 19).

In Table 3, the associations between different variables and neoplastic lesions were analyzed using the chi-square test and Fisher’s exact test. A significant association was found between the prevalence of neoplastic lesions and statins (C10AA), with individuals who reported taking statins having a lower prevalence of oral lesions. We observed that there is a significant association between the prevalence of mesenchymal neoplastic lesions and the use of antidiabetic medications. The prevalence of neoplastic lesions was higher in individuals who report taking antidiabetics. A significant association was found between the prevalence of non-neoplastic lesions and the use of antidiabetic medications, with the prevalence of non-neoplastic lesions being lower in individuals who report taking antidiabetics. Also, another association was found between mesenchymal neoplastic lesions and a history of oncological disease, with the prevalence of neoplastic lesions being higher in individuals reporting a history of oncological disease.

## 4. Discussion

This retrospective cross-sectional study evaluated oral lesions in patients seeking dental care at the EMDC in the metropolitan area of Lisbon, the most populous area in Portugal, with 2,871,133 inhabitants [17]. The EMDC is an important reference center for dental care in the Lisbon region, serving a diverse patient population.

Monteiro et al. [18] analyzed pathology reports of oral and maxillofacial lesions from 1990 to 2006 in the northern region. Due to methodological and sample differences, direct comparisons between these studies are unwarranted. The first study also excluded variables such as smoking, alcohol consumption, denture use, medication, and systemic diseases.

The primary objective of this study was to evaluate the occurrence of oral lesions among individuals attending EMDC. Overall, the prevalence of oral lesions in the studied population was 10.2%, which is similar to the findings of Oivio et al. [19] (10.5%). The prevalence in our study was higher than in the study by Saraswathi et al. [20] (4.1%) and lower than in studies by Al-Mobeeriek and AlDosari [21] (15%), Parlak et al. [22] (26.2%), Demko et al. [23] (26.7%), Jahanbani et al. [24] (28%), and Blanco et al. [25] (76.9%). Variations in prevalence rates may be influenced by region, country, demographic factors, sample size, and data source [26,27].

The study population consisted of 264 individuals, with 155 (58.7%) females and 109 (41.3%) males, a larger sample compared to studies such as Collins et al. [28] (n = 248), Kumar et al. [27] (n = 198), El Toum et al. [6] (n = 178), and Bajracharya et al. [29] (n = 111).

Non-neoplastic lesions were the most common in this population (75.0%), consistent with the findings of Collins et al. [28]. The most common lesion within the non-neoplastic diagnoses was fibroepithelial hyperplasia (36.7%), as in the study by Kelloway et al. [30]. Fibroepithelial hyperplasia was the most common lesion identified in this study, which was expected due to the susceptibility of the oral cavity to various traumatic and pathological stimuli. In our study, the proportion of patients using dental prostheses was high (33.7%), which is much higher than the results found in the study by Collins et al. [28] study (1.3%). Conversely, an overall higher prevalence of oral lesions has been reported in denture wearers [31,32]. Inflammatory odontogenic cyst was the second most common lesion, representing 7.2% of the total sample. This is in agreement with the study by Monteiro et al. [18], in which the odontogenic cyst was the most common lesion in the group of odontogenic tumors and cysts (8.4%). However, the prevalence may be underestimated because these lesions are often diagnosed clinically and radiographically without pathologic analysis. This is expected due to the high global and national prevalence of dental caries. More than one-third of the world’s population lives with untreated dental caries, making it the most common non-communicable disease and a major public health concern for populations and governments worldwide. The estimated global average prevalence of dental caries in permanent teeth is 29%, and the number of cases has reached more than 2 billion [10]. In Portugal, the prevalence of untreated dental caries in permanent teeth ranges from 23.3% to 30.6% [33].

The prevalence of potentially malignant oral diseases in this study was 11.0%, which is similar to the studies by Gupta et al. [34] (7.76%) and Campisi and Margiotta [31] (13.8%). However, this is higher than in the study by Kumar et al. [27] (5.63%) and lower than in the studies by Gambhir et al. [35] (22.2%) and Ferreira et al. [36] (29.6%).

In our study population, the prevalence of hyperkeratosis was 4.9%. This was the most common histopathological finding associated with oral leukoplakia. Several studies have reported a similar prevalence of leukoplakia, such as in the studies by Gupta et al. [34] (3.37%) and Kumar et al. [27] (3.1%). However, this was lower in the studies by Oivio et al. [19] (0.5%), Saraswathi et al. [20] (0.59%), Carrard et al. [37] (1.01%), Pentenero et al. [38] (1.15%), Ferreira et al. [36] (2.3%), Bhatnagar et al. [39] (2.83%), and Mehrotra et al. [40] (2.9%). And this was higher in studies by El Toum et al. [6] (5.1%) and Chung et al. [41] (7.44%). Warnakulasuriya [42] reported a higher prevalence of leukoplakia in smokers. This difference may be explained by the different study populations and smoking habits in Lebanon, India, and Taiwan.

Oral lichen planus had a prevalence of 4.9% in our study, which is lower than the study by Sixto-Requeijo et al. [43] (14.1%), higher than many reports [5,19,21,27,34,37,39,41], and higher than the global estimate of 1.01% [44]. Our patients with lichen planus were mostly non-smokers (76.9%), which confirms data from the literature showing a negative association between lichen planus and smoking [45].

The most common observed benign tumors were hemangiomas (4.5%) and squamous cell papilloma (4.5%). According to Léauté-Labrèze et al. [46], hemangioma is the most common benign neoplasm in childhood, appearing in the first weeks of life.

The malignant lesions of the oral cavity observed in our study were relatively low (0.8%) and seem to fit well within the various reports, ranging from 0.2% to 13.5% [25,26,27,29,34,35,40]. Two patients with a malignant oral neoplasm were identified. The most common malignancy was squamous cell carcinoma, which is consistent with several reports [30,43]. Among others, Chowdhury and Satoskar [47] studied a comparison of Japanese and Indian patients with oral carcinoma, and the results suggest that the pattern of demographic factors could have an influence on the development (or behavior) of oral cancer.

The data from our sample showed that oral lesions were most commonly observed in the age group of 45–64 years (38.3%). Two studies reported a lower prevalence of oral lesions in the younger group when compared to older individuals [6,48], which could be related to different habits acquired with age.

According to our study for gender distribution, there is a slightly higher number of biopsies in female patients (58.7%) compared to male patients (41.3%). This finding is consistent with previous reports [21,28,43] and can be explained by the fact that there is a larger female population in the region studied, according to the census of 2021 [49]. However, this is in contrast to other studies that found a higher prevalence of oral lesions in males [6,20,38,39]. This difference may be related to behavioral habits, such as the tendency to smoke and alcoholism in males, or the fact that males generally have a poorer health status than females, which also applies to oral health [50]. In our study, the most commonly biopsied site was the cheek mucosa (23.5%), followed by the gingiva (22.3%). These results are in agreement with the study by Bajracharya et al. [29].

Squamous cell carcinoma was found in the gingiva and tongue, which aligns with several studies that report a predominance of squamous cell carcinoma on the tongue [18,43].

In the present study, 26.5% were smokers, 0.4% were alcoholics, and 0.4% were drug users. It is well-documented that smoking and alcohol consumption are important risk factors for oral cancer and premalignant lesions [32,38,40,41,51,52]. The co-use of alcohol and tobacco has been shown to increase the risk of head and neck squamous cell carcinoma (HNSCC) by a factor of 40 [52]. Worldwide, tobacco or alcohol are responsible for 72% of HNSCC cases, with 35% attributable to both combined [53].

Regarding the occurrence and distribution of systemic diseases, our study showed that 34.5% of participants were hypertensive, 11.7% were diabetic, 11.7% were hypercholesterolemic, and 11.7% had a mental disorder. These results are consistent with those in other studies [6,54]. There are studies reporting a high prevalence of oral mucosal lesions in patients with diabetes mellitus [55,56]. Guggenheimer et al. [57] reported that this variable prevalence of oral lesions may reflect the different physiological behaviors of the two clinical types of diabetes.

In our study, 158 individuals (59.8%) were taking medication regularly. Of these, 33.7% reported using antihypertensive medications (C09), 15.2% used statins (C10AA), 12.1% used antidepressants (N06A), and 10.6% used antidiabetic medications (A10). In the study by Gheno et al. [54], the prevalence of drug use was 22.2% for antihypertensive medications and 3.9% for hypoglycemic medications. In the study by El Toum et al. [6], it was 10.7% for antihypertensives and 3.9% for antidiabetics. There were 84 individuals (53.2%) who were polymedicated.

In our study, there was a significant association between the prevalence of lesions and statins (C10AA), with the prevalence of lesions being lower in individuals who reported taking statins. The anticancer properties of statins have been the subject of several studies, and their potential as repurposed drugs for the treatment of cancer has been suggested, with several studies suggesting that statins have a number of beneficial anticancer effects [58,59]. However, in our study, most of the lesions were benign. Nevertheless, in addition to the lipid-lowering and anticancer properties of statins, they have been reported to have several promising effects on oral health, including chronic periodontitis [60], alveolar bone loss due to either extraction or chronic periodontitis [61,62], implant osseointegration [63], dental pulp cells [64], orthodontic tooth movement [65], tissue healing (wound/bone healing) [66], and salivary gland function [67]. However, the specific impact of statins on the development or progression of oral mucosal lesions remains underexplored. Given the observed association in our sample, future studies should aim to investigate this relationship in more detail, ideally through prospective designs with clinical correlation.

There was also an association between neoplastic lesions, and, among others, mesenchymal lesions and a history of oncologic disease. And we also observe that there is a significant association between the prevalence of mesenchymal neoplastic lesions and the use of antidiabetic medications. The prevalence of neoplastic lesions was higher in individuals who reported using antidiabetic medications. And there was a significant association between non-neoplastic lesions and the use of antidiabetic medications. However, the prevalence of non-neoplastic lesions was lower in individuals who reported using antidiabetic medications. The influence of antidiabetic medications on cancer risk and their dual effects, such as promoting and inhibiting cancer risk, have been discussed and remain a controversial topic. Studies have identified insulin and insulin secretagogues as hypoglycemic agents that may increase cancer risk. Conversely, insulin sensitizers appear to have a protective effect against cancer and may play a role in the prevention of oral squamous cell carcinoma in individuals with lichen planus [68,69]. In our study, the type of antidiabetic medication was not recorded, so we do not know whether the population studied was taking insulin or other antidiabetic medications.

As with any retrospective study, our research has limitations, particularly regarding former smokers, as we did not have data on the timing of smoking cessation. The use of multiple medications and frequent changes in treatment make it difficult to assess the effect of a specific medication on cancer risk. This study is an observational study of patients referred for oral health consultation, not an epidemiologic study. It estimates the prevalence in our subpopulation and includes pathologic diagnoses, providing more accurate results than studies based on clinical diagnoses alone. In addition, we included various sociodemographic, behavioral, and health factors, enhancing our understanding of the epidemiology of different oral lesions. It is worth noting that we evaluated only biopsied lesions; therefore, oral lesions that were not biopsied were not included. Our results are consistent with previous studies, adding more recent clinical information and relevant databases.

## 5. Conclusions

In this study, non-neoplastic oral lesions were the most commonly observed, while neoplastic lesions were more frequently found among older adults, particularly those in advanced age groups. The buccal mucosa and gingiva were identified as the most common sites for these lesions. A significant association was observed between the prevalence of neoplastic lesions and statin use, with neoplastic lesions being less common among statin users, which may suggest a potential protective effect worth investigating in future longitudinal studies. Additionally, a significant association was found between neoplastic and non-neoplastic lesions and the use of antidiabetic medications. Neoplastic lesions were more prevalent among individuals using antidiabetic medications, whereas non-neoplastic lesions were less frequent in this group. These findings raise important questions regarding the biological effects of antidiabetic therapies on oral tissues.

These data highlight the importance of integrating systemic health information, particularly pharmacological history, into oral health assessments. Further research is warranted to explore the underlying mechanisms behind these associations and to enhance the understanding of patient profiles. Comprehending the full spectrum of oral cavity lesions, their prevalence, and their associated risk factors is essential for developing more effective prevention strategies and mitigating disease progression. This highlights the need for multidisciplinary collaboration to ensure that patients receive comprehensive, evidence-based care. Despite its limitations, this study underscores the importance of well-structured public health initiatives aimed at educating the Portuguese population about the impact of oral lesions and their management.

## Figures and Tables

**Table 1:** Sociodemographic, behavioral, biometric, health, and oral hygiene data (n = 264).

Variable		n (%)
Gender	Female	155 (58.7)
	Male	109 (41.3)
Ager (years)	20–44	72 (27.3)
	45–64	101 (38.3)
	≥65	91 (34.5)
Employment status	Student	19 (7.2)
	Employed	117 (44.3)
	Unemployed	36 (13.6)
	Retired	92 (34.8)
Toothbrushing frequency	2–3 times/daily	205 (77.7)
	1 time/daily	50 (18.9)
	2–6 times/weekly	9 (3.4)
Smoking status	Smoker	70 (26.5)
	Non-smoker	194 (73.5)
Active smokers (Cigarettes per day) (n = 70)	Light (<10)	32 (45.7)
	Medium (10–20)	27 (38.6)
	Heavy (<20)	11 (15.7)
Systemic diseases	Yes	172 (65.2)
	No	92 (34.8)
History of oncological disease	Yes	13 (4.9)
	No	251 (95.1)
Medication	Yes	158 (59.8)
	No	106 (40.2)
Use of oral prosthesis	Yes	89 (33.7)
	No	175 (66.3)

**Table 2:** Oral lesions distribution as a function of gender, age, employment status, toothbrushing frequency, smoking, drugs, alcoholism, systemic diseases, history of oncological disease, medication, and use of oral prosthesis (n = 264).

Variates/Diagnostic Category		Non-Neoplastic Lesion (%)	Neoplastic Lesion (%)	Epithelial (%)	Mesenchymal (%)	Hematologic (%)
Gender	F	119 (60.1)	20 (48.8)	7 (43.8)	12 (50.0)	1 (100.0)
	M	79 (39.9)	21 (51.2)	9 (56.3)	12 (50.0)	0 (0.0)
Age	20–44	53 (26.7)	14 (34.1)	7 (43.8)	7 (29.2)	0 (0.0)
	45–64	81 (40.9)	9 (22.0)	3 (18.8)	6 (25.0)	0 (0.0)
	≥65	64 (32.3)	18 (43.9)	6 (37.5)	11 (45.8)	1 (100.0)
Employment status	Student	10 (5.1)	7 (17.1)	4 (25.0)	3 (12.5)	0 (0.0)
	Employed	96 (48.5)	11 (26.8)	4 (25.0)	7 (29.2)	0 (0.0)
	Unemployed	30 (15.2)	3 (7.3)	1 (6.3)	2 (8.3)	0 (0.0)
	Retired	62 (31.3)	20 (48.8)	7 (43.8)	12 (50.0)	1 (100.0)
Toothbrushing frequency	2–3 times/daily	151 (76.3)	31 (75.6)	8 (50.0)	22 (91.7)	1 (100.0)
	1 time/daily	40 (20.2)	8 (19.5)	7 (43.8)	1 (4.2)	0 (0.0)
	2–6 times/weekly	7 (3.5)	2 (4.9)	1 (6.3)	1 (4.2)	0 (0.0)
Smoking status	Smoker	53 (26.8)	10 (24.4)	4 (25.0)	6 (25.0)	0 (0.0)
	Non-smoker	145 (73.2)	31 (75.6)	12 (75.0)	18 (75.0)	1 (100.0)
Active smokers (Cigarettes per day)	Light (<10)	22 (41.5)	7 (70.0)	4 (100.0)	3 (50.0)	0 (0.0)
	Medium (10–20)	22 (41.5)	1 (10.0)	0 (0.0)	1 (16.7)	0 (0.0)
	Heavy (<20)	9 (17.0)	2 (20.0)	0 (0.0)	2 (33,3)	0 (0.0)
Drugs	Yes	3 (1.5)	0 (0.0)	0 (0.0)	0 (0.0)	0 (0.0)
	No	195 (98.5)	41 (100.0)	16 (100.0)	24 (100.0)	1 (100.0)
Alcoholism	Yes	0 (0.0)	0 (0.0)	0 (0.0)	0 (0.0)	0 (0.0)
	No	198 (100.0)	41 (100.0)	16 (100.0)	24 (100.0)	1 (100.0)
Systemic diseases	Yes	125 (63.1)	29 (70.7)	10 (62.5)	18 (75.0)	1 (100.0)
	No	73 (36.9)	12 (29.3)	6 (37.5)	6 (25.0)	0 (0.0)
History of oncological disease	Yes	7 (3.5)	5 (12.2)	1 (6.3)	4 (16.7)	0 (0.0)
	No	191 (96.5)	36 (87.8)	15 (93.8)	20 (83.3)	1 (100.0)
Medication	Yes	116 (58.6)	24 (58.5)	6 (37.5)	17 (70.8)	1 (100.0)
	No	82 (41.4)	17 (41.5)	10 (62.5)	7 (29.2)	0 (0.0)
Use of dental prosthesis	Yes	66 (33.3)	14 (34.1)	3 (18.8)	10 (41.7)	1 (100.0)
	No	132 (66.7)	27 (65.9)	13 (81.3)	14 (58.3)	0 (0.0)

**Table 3:** Associations between the 4 most prevalent medications, history of oncological disease, and neoplastic lesions.

		Neoplastic Lesion (%)	*p*-Value	Epithelial (%)	*p*-Value	Mesenchymal (%)	*p*-Value	Hematologic (%)	*p*-Value
Antihypertensive (C09)	No	16.0	0.768 *	6.3	0.830 *	9.7	0.621 *	0.0	-
	Yes	14.6		5.6		7.9		1.1	
Statins (C10AA)	No	17.4	0.046 *	7.1	-	9.8	0.549 **	0.4	-
	Yes	5.0		0.0		5.0		0.0	
Antidepressant (N06A)	No	15.5	1.00 **	6.5	0.702 **	8.6	0.508 **	0.4	-
	Yes	15.6		3.1		12.5		0.0	
Antidiabetics (A10)	No	12.7	0.001 **	5.1	0.075 **	7.2	0.007 **	0.4	-
	Yes	39.3		14.3		25.0		0.0	
History of oncological disease	No	14.3	0.035 **	6.0	0.565 **	8.0	0.022 **	0.4	-
	Yes	38.5		7.7		30.8		0.0	

* Chi-square test. ** Fisher’s exact test.

## Data Availability

The raw data supporting the conclusions of this article will be made available by the authors on request.

## Abbreviations

The following abbreviations are used in this manuscript:

EMDC	Egas Moniz Dental Clinic
HNSCC	Head and Neck Squamous Cell Carcinoma
HPV	Human Papillomavirus
SDGs	Sustainable Development Goals
WHO	World Health Organization
